# Wearable feedback systems for rehabilitation

**DOI:** 10.1186/1743-0003-2-17

**Published:** 2005-06-29

**Authors:** Michael Sung, Carl Marci, Alex Pentland

**Affiliations:** 1 The Media Laboratory, Massachusetts Institute of Technology, Cambridge, MA, USA; 2 Massachusetts General Hospital, Department of Psychiatry, Boston, MA, USA

## Abstract

In this paper we describe LiveNet, a flexible wearable platform intended for long-term ambulatory health monitoring with real-time data streaming and context classification. Based on the MIT Wearable Computing Group's distributed mobile system architecture, LiveNet is a stable, accessible system that combines inexpensive, commodity hardware; a flexible sensor/peripheral interconnection bus; and a powerful, light-weight distributed sensing, classification, and inter-process communications software architecture to facilitate the development of distributed real-time multi-modal and context-aware applications. LiveNet is able to continuously monitor a wide range of physiological signals together with the user's activity and context, to develop a personalized, data-rich health profile of a user over time. We demonstrate the power and functionality of this platform by describing a number of health monitoring applications using the LiveNet system in a variety of clinical studies that are underway. Initial evaluations of these pilot experiments demonstrate the potential of using the LiveNet system for real-world applications in rehabilitation medicine.

## Background and Introduction

Over the next decade, dramatic changes in healthcare systems are needed worldwide. In the United State's alone, 76 million baby boomers are reaching retirement age within the next decade [1]. Current healthcare systems are not structured to be able to adequately service the rising needs of the aging population, and a major crisis is imminent. The current system is dominated by infrequent and expensive patient visits to physician offices and emergency rooms for prevention and treatment of illness. The failure to do more frequent and regular health monitoring is particularly problematic for the elderly with multiple co-morbidities and often tenuous and rapidly changing health states. Even more troubling is the fact that current medical specialists cannot explain how most problems develop because they usually only see patients when something has already gone wrong.

Given this impending healthcare crisis, it is imperative to extend healthcare services from hospitals into home environments. Although there has been little success in extending health care into the home, there clearly is a huge demand. In 1997, Americans spent $27 billion on health care outside of the health care establishment, and that amount has been increasing [2]. Moreover, our dramatically aging population makes it absolutely necessary to develop systems that keep people out of hospitals. By 2030, nearly 1 out of 2 households will include someone who needs help performing basic activities of daily living and labor-intensive interventions will become impractical because of personnel shortage and cost [2].

The best solution to these problems lies in more proactive healthcare technologies that put more control into the hands of patients. The vision is a healthcare system that will help an individual to maintain their normal health profile by providing better monitoring and feedback, so that the earliest signs of health problems can be detected and corrected. This can be accomplished affordably by continuously monitoring a wide range of vital signals, providing early warning systems for people with high-risk medical problems, and "elder care" monitoring systems that will help keep seniors out of nursing homes and in their independent living arrangements.

Most available commercial mobile healthcare platforms have focused on data acquisition applications, with little attention paid to enabling real-time, context-aware applications. Companies such as VivoMetrics [3], Bodymedia [4], and Mini-Mitter [5], have extended the basic concept of the ambulatory Holter monitor (enabling a physician to record a patient's ECG continuously for 24–48 hours), which for three decades has been the only home health monitor with widespread use [6]. Additions to this individual monitoring paradigm have been extended along two fronts: medical telemetry and real-time critical health monitoring. Regarding the former, various inpatient medical telemetry systems have been developed in recent years, focusing on providing an infrastructure for transporting and storing data from the patient to caregivers for later analysis [7]. In terms of the latter, a few systems have extended the health monitoring concept by augmenting a physiological monitor (usually based on a single physiological sensor) with specialized algorithms for real-time monitoring within specific application domains, such as heart arrhythmia, epileptic seizures, and sleep apnea, which can potentially trigger alerts when certain critical conditions or events occur [8,9]. However, the development of proactive healthcare technologies beyond these basic telemedicine and individual event monitoring applications has been rather slow. The main limitation has been the large costs and inflexibility of limited monitoring modalities associated with these technologies and the impracticality for long-term use in general settings.

This paper presents LiveNet, a flexible distributed mobile platform that can be deployed for a variety of proactive healthcare applications that can sense one's immediate context and provide feedback. Based on cost-effective commodity PDA hardware with customized sensors and data acquisition hub plus a lightweight software infrastructure, LiveNet is capable of local sensing, real-time processing, and distributed data streaming. This integrated monitoring system can also leverage off-body resources for wireless infrastructure, long-term data logging and storage, visualization/display, complex sensing, and computation-intensive processing. The LiveNet system allows people to receive real-time feedback from their continuously monitored and analyzed health state. In addition, LiveNet can communicate health information to caregivers and other members of an individual's social network for support and interaction. Thus, by combining general-purpose commodity hardware with specialized health/context sensing within a networked environment, it is possible to build a multi-functional mobile healthcare device that is at the same time a personal real-time health monitor, multimodal feedback interface, context-aware agent, and social network support enabler and communicator.

With the development of increasingly powerful diagnostic sensing technology, doctors can obtain more context specific information directly, instead of relying on a patient's recollection of past events and symptoms, which tend to be vague, incomplete, and error prone. While many of these specialized sensing technologies have improved with time, most medical equipment is still a long way off from the vision of cheap, small, mobile, and non-invasive monitors. Modern imaging technology costs thousands of dollars per scan, requires room-sized equipment chambers, and necessitates uncomfortable and time-consuming procedures. Personal health systems, on the other hand, must be lightweight, easy-to-use, unobtrusive, flexible, and non-invasive to make headway as viable devices that people will use.

As such, there is tremendous potential for basic non-invasive monitoring as a complement to more invasive diagnostic sensing devices. The LiveNet system focuses on using combinations of non-invasive sensing and contextual features (for example, heart rate, motion, voice features, skin conductance, temperature/heat flux, location) that can be correlated with more involved clinical physiology sensing such as pulse oximetry, blood pressure, and multi-lead ECG. Sensors in the LiveNet system can continuously monitor autonomic physiology, motor activity, sleep patterns, and other indicators of health. The data from these sensors can then be used to build a personalized profile of performance and long-term health over time tailored to the needs of the patient and their healthcare providers. This unique combination of features also allows for quantification of personal contextual data such as amount and quality of social interactions and activities of daily living. This type of information is potentially very useful for increasing the predictive power of diagnostic systems.

The most important aspect of the LiveNet system is that it enables practical, long-term, context specific continuous monitoring. Continuous monitoring ensures the capture of relevant events and the associated physiology wherever the patient is, expanding the view of healthcare beyond the traditional outpatient and inpatient settings. Long-term monitoring has the potential to help create new models of health behavior. For example, long-term monitoring may provide important insights into the efficacy and effectiveness of medication regimes on the physiology and behavior of a patient over time at resolutions currently unobtainable. In addition, progress in terms of understanding human physiology and behavior will result from the fact that long-term trends can be explored in detail. Such advances include tracking the development and evolution of diseases, development of predictors of response to treatment and relapse prevention, monitoring changes in physiology as people grow older, comparing physiology across different populations (gender, ethnicity, etc), and even knowing characteristic physiology patterns of people who are *healthy *(this last example is particularly important when it is necessary as a diagnostic methodology designed to quantitatively define abnormal behavior). The goal is to be able to detect repeating patterns in complex human behavior by analyzing the patterns in data collected from the LiveNet system. From continuous monitoring, a very fine granularity of quantitative data can be obtained, in contrast to the surveys and history-taking that has been the mainstay of long-term studies and health interventions to date.

## The LiveNet System

There are three major components to the LiveNet system: a personal data assistant (PDA) based mobile wearable platform, the software network and resource discovery application program interface (API), and a real-time machine learning inference infrastructure. The LiveNet system demonstrates the ability to use standardized PDA hardware tied together with a flexible software architecture and modularized sensing infrastructure to create a system platform where sophisticated distributed healthcare applications can be developed. While the current system implementations are based on PDAs, the software infrastructure is designed to be portable to a variety of mobile devices, including cell phones, tablet computers, and other convergence devices. As such, the system leverages commercial off-the-shelf components with standardized base-layer communication protocols (e.g., TCP/IP); this allows for the rapid adoption and deployment of these systems into real-world settings.

The LiveNet system is based on the MIThril wearable architecture developed at the Massachusetts Institute of Technology (MIT) Media Laboratory [10]. This proven architecture combines inexpensive commodity hardware, a flexible sensor/peripheral interconnection bus, and a powerful light-weight distributed sensing, classification, and inter-process communications software layer to facilitate the development of distributed real-time multi-modal and context-aware applications.

The LiveNet hardware and software infrastructure provides a flexible and easy way to gather heterogeneous streams of information, perform real-time processing and data mining on this information, and return classification results and statistics. This information can result in more effective, context-aware and interactive applications within healthcare settings.

A number of key attributes of the LiveNet system that make it an enabling distributed healthcare system include:

• Hierarchical, distributed modular architecture

• Based on standard commodity/embedded hardware that can be improved with time

• Wireless capability with resource posting/discovery and data streaming to distributed endpoints

• Leverages existing sensor designs and commercial sensors for context-aware applications that can facilitate interaction in a meaningful manner and provide relevant and timely feedback/information

• Unobtrusive, minimally invasive, and minimally distracting

• Abstracted network communications with secure sockets layer (SSL) encryption with real-time data streaming and resource allocation/discovery

• Continuous long-term monitoring capable of storing a wide range of physiology as well as contextual information

• Real-time classification/analysis and feedback of data that can promote and enforce compliance with healthy behavior

• Trending/analysis to characterize long-term behavioral trends of repeating patterns of behavior and subtle physiological cues, as well as to flag deviations from normal behavior

• Enables new forms of social interaction and communication for community-based support by peers and establishing stronger social ties within family groups

### LiveNet Mobile Technology

The LiveNet system is currently based on the Sharp Zaurus (Sharp Electronics Corporation, U.S.A.), a Linux-based PDA mobile device that leverages commercial development and an active code developer community. Although LiveNet can utilize a variety of Linux-based devices, the Zaurus PDA provides a very convenient platform. This device allows applications requiring real-time data analysis, peer-to-peer wireless networking, full-duplex audio, local data storage, graphical interaction, and keyboard/touch screen input.

In order to effectively observe contextual data, a flexible wearable platform must have a means to gather, process, and interpret this real-time contextual data [34]. To facilitate this, the LiveNet system includes a modular sensor hub called the Swiss-Army-Knife 2 (SAK2) board that can be used to instrument the mobile device for contextual data gathering.

The SAK2 is a very flexible data acquisition board that serves as the central sensor hub for the LiveNet system architecture. The SAK2 incorporates a powerful 40 MHz PIC microcontroller, high efficiency regulated power (both 5 V and 3.3 V to power the board and sensor network) from a flexible range of battery sources, a 2.4 GHz wireless tranceiver capable of megabit data rates, compact flash based memory storage, and various interface ports (I2C, RS-232 serial, daughter board connector).

The SAK2 board was designed primarily to interface a variety of sensing technologies with mobile device-based wearable platforms to enable real-time context-aware, streaming data applications. The SAK2 is an extremely flexible data acquisition hub, allowing for a wide variety of custom as well as third-party sensors to interface to it. In addition to being a sensor hub, the SAK2 can also operate in stand-alone mode (i.e., without a Zaurus or mobile PDA host) for a variety of long-term data acquisition (using the CF card connector) and real-time interactive applications.

### Physiologic and Contextual Sensing Technology

In order to support long-term health monitoring and activities of daily living applications, a specialized extensible, fully integrated physiological sensing board called the BioSense was developed as a special add-on board to the SAK2. The board incorporates a three dimensional (3D) accelerometer, ECG, EMG, galvanic skin conductance, a serial-to-I2C converter (which can allow the simultaneous attachment of multiple 3^rd ^party serial-based sensing devices to the sensor network), and independent amplifiers for temperature/respiration/other sensors that can be daisy-chained to provide a flexible range of amplification for arbitrary analog input signals. Toward developing more non-invasive sensing technologies, we have started a collaboration with the Fraunhofer Institute to shrink the BioSense hardware to create a microminiaturized embedded system that can be incorporated in wearable fabrics. A prototype of a working lead-less lightweight ECG shirt based on conductive textiles has already been created.

Along with the core physiological sensing capabilities of the LiveNet system with BioSense daughter board, a whole host of other custom and third party sensors can be seamlessly integrated with the system, including:

• Wearable Multiple Sensor Acquisition (WMSAD) Board, providing a 3D accelerometer, infrared (IR) tag, IR tag readers (vertical, for in-door location in place of GPS, and horizontal for peer or object identification), and microphone for telephony-grade 8-kHz audio. This is interfaced to the SAK2 via the I2C port. [11].

• Squirt IR Tags: IR beacons that can broadcast unique identifiers (up to 4 independent signals from separately mounted and direction-adjustable IR-LEDS) [12]. These can be used to tag individuals, objects, locations (such used in arrays on the ceiling to identify location to within meter resolution within indoor settings where GPS is not effective), or even as environmental sensors to identify the actuation of certain events such as opening/closing of drawers, cabinets, or doors.

• IR Tag Reader: to be used in conjunction with the Squirt tags to be able to identify tagged objects, people, or even locations [12].

• Accelerometer Board: 3D accelerometer board very useful for a variety of activity classification. It has been demonstrated that a single accelerometer board can be used to accurately classify activity state (standing, walking, running, lying down, biking, walking up stairs, etc). Interfaced to the SAK2 using the sensor port.

• BodyMedia SenseWear: An integrated health sensor package which provides heart rate (via a Polar heart strap), galvanic skin response, 2D accelerometer, temperature (ambient and skin), and heat flux in a small form-factor package worn on the back of the arm [4]. The SAK2 can interface to the SenseWear wirelessly via a 900-MHz tranceiver attached to the serial-toI2C bridge (the tranceiver interface and heart rate monitor was discontinued in the SenseWear Pro 2)

• MITes Environmental Sensor: a wireless 3D accelerometer using the nRF 2.4 GHz protocol has been developed by the house_n group at the Media Lab for wireless environmental sensors for monitoring human activities in natural settings [13]

• Socio-Badges: Multifunctional boards with on-board DSP processor capable of processing audio features, RF tranceiver, IR transceiver, a brightness-controllable LED output display, vibratory feedback, navigator switch, flash memory, audio input/microphone and optional LCD display [14]. This badge is meant for social-networking experiments and other interactive distributed applications.

The sensor hub also allows us to interface with a wide range of commercially available sensors, including pulse oximetry, respiration, blood pressure, EEG, blood sugar, humidity, core temperature, heat flux, and CO_2 _sensors. Any number of these sensors can be combined through junctions to create a diversified on-body sensor network. The LiveNet system can also be outfitted with BlueTooth, Secure Data (SD), or Compact Flash (CF) based sensors and peripherals, and other I/O and communication devices including GSM/ GPRS/ CDMA/ 1 × RTT modems, GPS units, image and video cameras, memory storage, and even full-VGA head-mounted displays.

With the combined physiological sensing board and third-party sensors, a fully outfitted LiveNet system can simultaneously and continuously monitor and record 3D accelerometer, audio, ECG, EMG, galvanic skin response, temperature, respiration, blood oxygen, blood pressure, heat flux, heart rate, IR beacon, and up to 128 independently channeled environmental activity sensors. The sensor data and real-time classification results from a LiveNet system can also be streamed to off-body servers for subsequent processing, trigger alarms or notify family members and caregivers, or displayed/processed by other LiveNet systems or computers connected to the data streams for complex real-time interactions.

### Software

The software architecture allows designers to quickly design distributed, group-based applications that use contextual information about the members of a group. Layered on top of standard libraries, this middleware comprises three important parts: the Enchantment Whiteboard, the Enchantment Signal system, and the MIThril Real-Time Context Engine [10]. Respectively, these three layers provide the ability to easily coordinate between distributed applications, transmit high bandwidth signals between applications, and create classification modules that make a group's changing contextual information available to applications.

The Enchantment Whiteboard system is a distributed, client/server, inter-process communication system that provides a lightweight way for applications to communicate. This system processes, publishes, and receives updates, decoupling information from specific processes. This is particularly useful in mobile, group based applications where group members may not be known a priori and may come and go over time.

For higher bandwidth signals, especially those related to the sharing and processing of sensor data for context aware applications, we developed the Enchantment Signal system. The Signal system is intended to facilitate the efficient distribution and processing of digital signals in a network-transparent manner. The Signal system is based on point-to-point communications between clients, with signal "handles" being posted on Whiteboards to facilitate discovery and connection. In the spirit of Whiteboard interactions, the Signal API abstracts away any need for signal produces to know who, how many, or even if, there are any connected signal consumers.

The MIThril Real-Time Context Engine is an open-source, lightweight, and modular architecture for the development and implementation of real-time context classifiers for wearable applications. Using the context engine, we can implement lightweight machine learning algorithms (capable of running on an embedded system like the Zaurus PDA) to process streaming sensor data, allowing the systems to classify and identify various user-state context in real-time.

## Sample Applications

In the following section, a number of real-world case examples of clinical applications built upon various parts of the LiveNet system are detailed. These examples demonstrate the modular, configurable nature of the LiveNet infrastructure and the flexibility of the architecture to accommodate a variety of high bandwidth, real-time applications.

## Health and Clinical Classification

The LiveNet system has proven to be a convenient, adaptable platform for developing real-time monitoring and classification systems using a variety of sensor data, including accelerometer-based activity-state classification that can differentiate between a variety of activities (for example, running, walking, standing, biking, climbing stairs) [15], accelerometer-based head-nodding/shaking agreement classifiers, GSR-based stress and emotional arousal detectors, and audio-based speech feature classifiers that can help characterize conversation dynamics (for example, talking time, prosody, stress) [16].

Work on these real-time classifiers has also been extended to include a variety of health conditions. Examples of current collaborations between the MIT Wearable Computing Group [17] and healthcare providers have lead to a variety of pilot studies including a hypothermia study with the United States Natick Army Laboratories in Natick; a study on the effects of medication on the dyskinesia state of Parkinson's patients with neurologists at Harvard Medical School; a pilot epilepsy classifier study with the University of Rochester Center for Future Health; and a study of the course of depression treatment with psychiatrists at Harvard Medical School.

### Critical Soldier Monitoring

Army Rangers and other soldiers must perform physically and mentally demanding tasks under challenging environmental conditions ranging from extreme heat to extreme cold. Thermoregulation, or the maintenance of core body temperature within a functional range, is critical to sustained performance. A research collaboration with the Army Research Institute for Environmental Medicine (ARIEM) at the Army Natick Labs was initiated to study the effects of harsh environments on soldier physiology through the use of non-invasive sensing. Specifically, non-invasive accelerometer sensing was used to determine hypothermia and cold exposure state, as part of a broader initiative to develop a physiologic monitoring device for soldiers under the US. Army's Objective Force Warrior Program.

In the study, a real-time wearable monitor was developed using the LiveNet system that is capable of accurately classifying shivering motion through accelerometer sensing and analysis using statistical machine learning techniques [18]. Real-time working classifier systems were developed from Gaussian Mixture Models using frequency features derived from calculating a finite Fourier transform (FFT) on the raw accelerometer data. Preliminary data demonstrate that shivering can be accurately distinguished with up to 95% accuracy from general body movements in various activities using continuous accelerometer sensing. Results also indicate that specific modes of shivering (subjects in the study all exhibited a light shiver at a characteristic frequency at the start of the protocol that progressed into a more noisy and energetic shivering response spread across more frequency bands, and ending in a dampened shivering toward the end of the protocol) may correlate with core body temperature regimes, as a person is exposed to cold over time. In fact, preliminary results from six subjects show that we can triage a soldier into three core body temperature regimes (Baseline/Cold/Very Cold) with accuracies in the 92–98% range using HMM (Hidden Markov Models) modeling techniques. HMM modeling has the advantage of being able to accurately model the time-dependent changes in shivering over time as an individual is exposed to cold. This exploratory research shows promise of eventually being able to develop robust real-time health monitoring systems capable of classifying cold exposure of soldiers in harsh cold environments with non-invasive sensing and minimal embedded computational resources.

### Parkinson's Disease Monitoring

LiveNet promises to be especially effective for monitoring medical treatments. Currently, doctors prescribe medications based on population averages rather than individual characteristics, and they check the appropriateness of the medication levels only occasionally. With such a data-poor system, it is not surprising that medication doses are frequently over- or underestimated and that unforeseen drug interactions can occur. Stratifying the population into phenotypes using genetic typing will improve the problem, but only to a degree and only in limited ways currently.

Continuous monitoring of physiologic and behavioral parameters may be extremely effective in tailoring medications to the individual Parkinson's patient. In Parkinson's patients, there are a variety of symptoms and motor complications that can occur, ranging from tremors (rhythmic involuntary motions), akinesia (absence or difficulty in producing motion), hypokinesia (decreased motor activity), bradykinesia (slow down of normal movement), and dyskinesia (abnormal or disruptive movements). For these patients to function at their best, medications must be optimally adjusted to the diurnal variation of these symptoms. In order for this to occur, the managing clinician must have an accurate picture of how a patient's symptoms fluctuates throughout a typical day's activities and cycles. In these situations, a patient's subjective self-reports are not typically very accurate, so objective clinical assessments are necessary.

An automated Parkinson symptom detection system is needed to improve clinical assessment of Parkinson's patients. To achieve this, Dr. Klapper, from the Harvard Medical School, combined the LiveNet system's wearable accelerometers with neural network algorithms to classify the movement states of Parkinson's patients and provide a timeline of how the severity of the symptoms and motor complications fluctuate throughout the day [19,20]. Two pilot studies were performed, consisting of seven patients, with the goal of assessing the ability to classify hypokinesia, dyskinesia, and bradykinesia based on accelerometer data, clinical observation (using standard clinical rating scales), and videotaping. Using the clinical ratings of a patient as the gold standard, the result was highly accurate identification of bradykinesia and hypokinesia. In addition, the studies classified the two most important clinical problems – predicting when the patient "feels off" or is about to experience troublesome dyskinesia – with nearly 100% accuracy. Future collaborations will focus on integrating the physiologic responses in an effort to identify predictors of relapse in addition to the motion data in Parkinson's patients.

### Epilepsy Seizure Detection

A pilot study has also been initiated with the University of Rochester's Strong Hospital [21] to characterize and identify epileptic seizures through accelerometry and to begin to develop an ambulatory monitor with a real-time seizure classifier using the LiveNet system. Typically, epilepsy studies focus on EEG and EMG-based physiology monitoring. However, as demonstrated by the Parkinsons' and activity classification studies, accelerometry is a very powerful context sensor that can be applied to the domain of epilepsy. The study protocol is currently being designed, and we hope to have subject run in the Fall of 2005.

Of particular note to patients who have epilepsy is the fact that it can manifest itself in an extremely wide range of idiosyncratic motions, in contrast to Parkinsons' patients, whose movements typically follow distinct, characteristic motions. However, motions from the epileptic seizures of a particular individual are normally fairly consistent. As such, a motion classification system specifically tailored to a particular individual could be highly effective at being able to identify an epileptic seizure at onset and at sub-threshold levels of awareness. In addition, many times, the epileptic individual has no recollection of a seizure, so a system that could determine if a seizure has occurred could be very useful for doctors to be able to properly diagnose the type and pattern of epilepsy in patients or to develop applications to alert caregivers to changes that could lead to medication adjustments earlier in the course of the illness. Again, future directions will involve continuous physiologic and voice feature analysis in combination with the motion sensors to increase the accuracy and understanding of patients with epilepsy.

### General Activity Classification

Being able to predict an individual's immediate activity state is one of the most useful sources of contextual information. For example, knowing whether a person is driving, sleeping, or exercising could be useful for a health wearable to calculate general energy expenditure or to initiate an action. Many studies on activity classification have been conducted because of the importance to context-aware systems. Most previous studies on accelerometer-based activity classification that involves multiple activities states focuses on using multiple accelerometers and requires the specific placement of sensors on different parts of the body. In one study, it was shown that it is possible to obtain activity classification with an average of 84% for 20 daily activities (such as vacuuming, eating, folding laundry, etc), with the additional finding that classification accuracy dropped only slightly by decreasing the number of sensors to two including the wrist and waist) [22].

In contrast, we have conducted a pilot study on the use of the minimum set of sensors required to accomplish accurate activity classification. The ultimate goal is to use only a single sensor in random orientation placed close to a person's center of mass (i.e., near waist level), as this replicates the minimum setup requirements of a sensor-enabled mobile phone in the pocket of an individual. The goal is to demonstrate that accurate activity classification can be performed without the need for an extreme level of instrumentation (for example, some systems use up to 30 sensors [23]) or particular delicacy in the setup in order to achieve good classification results. This way, we are able to potentially reduce the cost of a recognition system as well as reducing the overall burden when using the technology.

Using a LiveNet system we have been able to discriminate between a set of major activities (for example, lying down, walking, running, sitting in the office, watching TV, and walking up/down stairs) with classification results in the 80–95% accuracy range using only a single accelerometer located on the torso of an individual [15]. This research is important as it indicates that it is feasible to do activity classification on embedded hardware without any specialized setup, wires, or other unwieldy parts. By integrating the accelerometer into an existing device that people are comfortable carrying around (for example, a cell phone), we can significantly lower the bar for developing a practical activity classification system to the mass market that is completely transparent to the user. When combined with physiological measurements such as heart rate and breathing rate, these measurements can then be collected to build a personalized profile of your body's performance and your nervous system's activation throughout your entire day, and assembled over a period of months or years to show long-term changes in overall cardiac fitness. In the future, computer software 'agents' (automatic computer programs) could even give you gentle reminders to keep up your routine if your activity level started to decline and make suggestions to optimize your performance.

### Depression Therapy Trending

Mental diseases rank among the top health problems worldwide in terms of cost to society. Major depression, for instance, is the leading cause of disability worldwide and in the U.S. Depressive disorders affects approximately 19 million American adults and has been identified by both the World Health Organization and the World Bank as the second leading cause of disability in the United States and worldwide [28,29].

Toward understanding the long-term biology associated with severe depression, we have recently initiated a pilot study to assess the physiological and behavioral responses to treatment in major depression in subjects in an inpatient psychiatric unit prior to, during, and following electroconvulsive therapy (ECT). This study, the first of its kind, intends to correlate basic physiology and behavioral changes with depression and mood state through a 24-hour, long-term, continuous monitoring of clinically depressed patients undergoing ECT. We are using non-invasive mobile physiologic sensing technology in combination with sensing devices on the unit to develop physiological and behavioral measures to classify emotional states and track the effects of treatment over time. This project is a joint collaboration with the Massachusetts General Hospital (MGH) Department of Psychiatry.

The goal of this study is to test the LiveNet system based on the known models of depression and prior clinical research in a setting with combined physiologic and behavioral measures with continuous ambulatory monitoring. It is anticipated that changes in these measures (namely, GSR response, hear rate/heart rate variability, motor activity, vocal features, and movement patterns) will correlate with improvements in standard clinical rating scales and subjective assessment following treatment for depression throughout the course of hospitalization. In the future, these correlates may be used as predictors of those patients most likely to respond to ECT, for early indicators of clinical response, or for relapse prevention.

The collaboration with MGH will serve to establish the LiveNet system's capabilities for engaging in significant long-term ambulatory clinical studies. The implications and clinical significance of the proposed research are broad. The development and refinement of a methodology that objectively and accurately monitors treatment response in major depression has implications for the diagnosis, treatment, and relapse prevention. Once a reliable index of physiologic and behavioral metrics for depression has been established, other environments outside of the inpatient setting become potential targets for assessment. The methodology developed also has the potential to help in the assessment, early diagnosis, and treatment prediction of other severe psychopathologies that have likely physiologic correlates and involve difficulty with social interactions. These include communication disorders and pervasive developmental disorders in children as well as the pre-clinical assessment of severe psychotic, mood, anxiety, and personality disorders. As our understanding of the central nervous system control of autonomic arousal improves and the neurobiology of depression continues to be discovered, future questions about the subtypes of depression and endophenotyping for genetic studies of depression can be studied in the ambulatory setting. This will lead to more sophisticated neurobiologic models of the mechanism of healing and ultimately to increased efficiency and efficacy of treatment.

### Quantifying Social Engagement

Social interaction is a complex and ubiquitous human behavior involving attitudes, emotions, nonverbal and verbal cues, and cognitive function. Importantly, impairment in social function is a hallmark for nearly every diagnostic category of mental illness including mood and anxiety disorders as well as dementia, schizophrenia, and substance abuse [24]. In addition, social isolation can be a significant stress for patients undergoing rehabilitation from surgical and medical procedures and illnesses. Thus, an important challenge for our behavior modeling technology is to build computational models that can be used to predict the dynamics of individuals and their social interactions.

Using LiveNet, we can collect data about daily interactions with family, friends, and strangers and quantify information such as how frequent are the interactions, the dynamics of the interactions, and the characteristics of such interactions using simple infrared (IR) sensors and IR tags to identify individuals. Using simple voice features (such as talking/non-talking, voice patterns, and interactive speech dynamics measures) derived from microphones, we can obtain a variety of useful social interaction statistics. We can even model an individual's social network and how that network changes over time by analyzing statistical patterns of these networks as they evolve [25]. Data on social function can be used as both a marker of improvement or rehabilitation progress or as an indicator of relapse and for use in relapse prevention.

## Long-Term Behavior Modeling and Trending

The LiveNet platform also lends itself naturally to be able to do a wide variety of long-term healthcare monitoring applications for physiological and behavioral trends that vary slowly with time by using the currently available physiological sensors. This has important implications for rehabilitation medicine. The ambulatory physiological and contextual sensing and the health classifiers discussed in Section 3.1 can be combined together in a hierarchical manner to develop time-dependent models of human behavior at longer timescales. Current systems are purely reactive (e.g. sounding an alarm after a person has a heart attack or falls down), and are dependent on classifying and determining in real-time when certain events have occurred.

While this type of application is very useful and potentially-life saving, these systems typically do not have any sense of the history of an individual and can only react to instantaneous events. By combining long-term trending with multimodal analysis, it is possible to develop more proactive systems and personalized data that can be used to catch problems before they manifest themselves (e.g. instead of reacting to a heart attack, one can predict beforehand that a heart attack is imminent). However, a proactive system requires more resources, as it must have context-aware and inference capabilities to be able to determine what the right information is to be directed at the right people, to the right places, at the right times, and for the right reasons. While challenging, small advances have been made in this regard.

In order to fully accomplish the goal of preventive monitoring, large databases in living situations are needed. The LiveNet system provides a convenient infrastructure to implement and rapidly prototype new proactive healthcare applications in this domain. It is very important from the proactive healthcare point of view that these individual classification systems also be able to determine trends in physiological/contextual state over time to provide not only immediate diagnostic power but also prognostic insight. We are collaborating on the MIT/TIAX PlaceLab, a cross-institutional research smart living environment [26], to provide a very robust infrastructure to be able to collect and study long-term health information in conjunction with data collected by LiveNet systems. We also have a collaboration with British Telecom to use LiveNet technology in similar long-term naturalistic home monitoring applications for eldercare.

The information collected from the multimodal sensors can then be used to construct activities of daily living, important information in being able to profile a person's healthy living style. Furthermore, these activities of daily living can initiate action on the part of the wearable PDA. Examples include experience sampling, a technique to gather information on daily activity by point of querying (which can be set to trigger based on movement or other sensed context by the PDA). The system can also proactively suggest alternative healthy actions at the moment of decision, where it has been demonstrated as being more effective at eliciting healthy behavior [27].

## Real-Time Multimodal Feedback Systems in Rehabilitation

An obvious domain for LiveNet is in physiology monitoring with real-time feedback and classification. The dominant healthcare paradigm that exists is to stream physiology data from an individual to a centralized server, where the higher-power processing and data visualization could be performed to post-process the data. The wearable system served mainly as a data acquisition vehicle, with little feedback or interaction capabilities. Now, it is possible for significant localized processing as well as displaying the result, which will open up the door to real-time interactive health applications. In fact, commercial systems are just beginning to incorporate these types of increased functionality.

It is possible to use a system such as LiveNet to go a step farther and demonstrate that mobile systems are capable of significant local processing for real-time feature extraction and context classification as well as provide the distributed wireless infrastructure for streaming information between systems, all on commodity hardware that is commonplace and available today. This will enable the real-time classification of medical conditions without the need for other infrastructure, available wherever the individual goes. The distributed nature of LiveNet can also allow systems to stream raw physiology or its combination with derived metadata/context very easily to any specified source(s), whether it is other mobile systems, data servers, or output displays such as projections.

By providing local processing capabilities, the time that is required to receive feedback for relevant health events is dramatically reduced. Historically, the time delay required to receive feedback can potentially take weeks, and be both problematic because of the iterative nature of determining the optimized treatment path. For people who are on medication or embarking on a prolonged rehabilitation schedule, for example, this delay in the feedback loop is particularly onerous. A doctor will recommend a dosage and medication regimen to try out, and the person goes home and tries the medication schedule for a while. If the person does not respond favorably to this drug schedule, they have to reschedule an appointment with the doctor, go in, and potentially take more tests, before getting a recommendation on a new schedule (such is the case for people with thyroid conditions, for example). This same scenario is also true with lengthy rehabilitation programs such as cardiac rehabilitation. This results in a very time consuming process as well as a significant drain on healthcare resources. In addition to the fact that the feedback loop can be very long in duration, the doctor is literally in the dark about the efficacy of the treatment, and so an iterative trial-and-error process is required.

Using the LiveNet system, it is possible to effectively reduce the time delay to process and receive health feedback. This is particularly true when the doctor can be either removed from the equation or visit length and frequency can be reduced, such as with real-time diagnostic systems that can provide effectively instantaneous classification on health state and context. Given that medication compliance is a major healthcare issue, especially among the elderly, with estimated costs of upwards of $100 billion annually [30], systems that can help remind and support compliance with appropriate feedback will help to promote healthy, preventive behavior.

Also, potential advances in more personalized medication and rehabilitation scheduling can be improved based on measured, quantitative physiological symptoms and behavioral responses, not based only on time scheduled approximations as the current practice. The effects of medication and rehabilitation treatment can be logged and recorded quantitatively and compared to changes in physiology, eventually with the goal of developing a real-time monitoring and drug delivery system. Future research will extend this work by developing real-time, closed-loop systems that can track the effects of individualized treatment over time.

Time-stamping of relevant events (either simple events or elaborated notes) in order to correlate these events with accurate continuous physiology and behavior data in an ambulatory setting also offers great potential. From this health information, real-time correlations to specific medical conditions as well as predictions of adverse outcomes can be made. This also has a potential impact in the research of physiology in the domain of clinical medication and intervention research, providing a streamlined path for Electronic Data Capture (EDC), where accurate reporting is an issue and human transcription errors and recall bias from surveys can be reduced. Potential benefits provided by continuous monitoring include automated and real-time data capture from patients for accurate reporting, feedback and notification for enforcing medication compliance in patients, assessment of the degree of medication compliance, removal of human error inherent in manual transcription/data entry, high-resolution time-stamping for accurate temporal characterization of events, and the ability to accurately correlate quantitative physiologic data to events for diagnosis and characterization

## Conclusion

The ability to provide new wearable technology for medical and surgical rehabilitation services is emerging as an important option for clinicians and patients. Wearable technology provides a convenient platform to be able to quantify the long-term context and physiological response of individuals. This, in turn, will support the development of individualized treatment systems with real-time feedback to help promote proper behavior. The ultimate goal of the research is to eventually be able to use LiveNet for developing practical monitoring systems and therapeutic interventions in ambulatory, long-term use environments.

**Figure 1:**
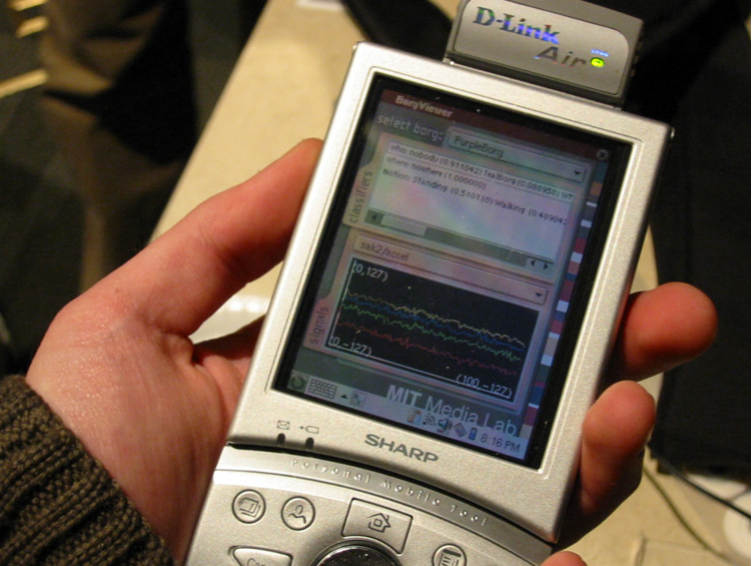
LiveNet wearable performing real-time FFT analysis and activity classification on accelerometer data, visualizing the results, as well as wirelessly streaming real-time ECG/GSR/temperature and classification results to a remote computer with a projection display as well as peer LiveNet systems.

**Figure 2:**
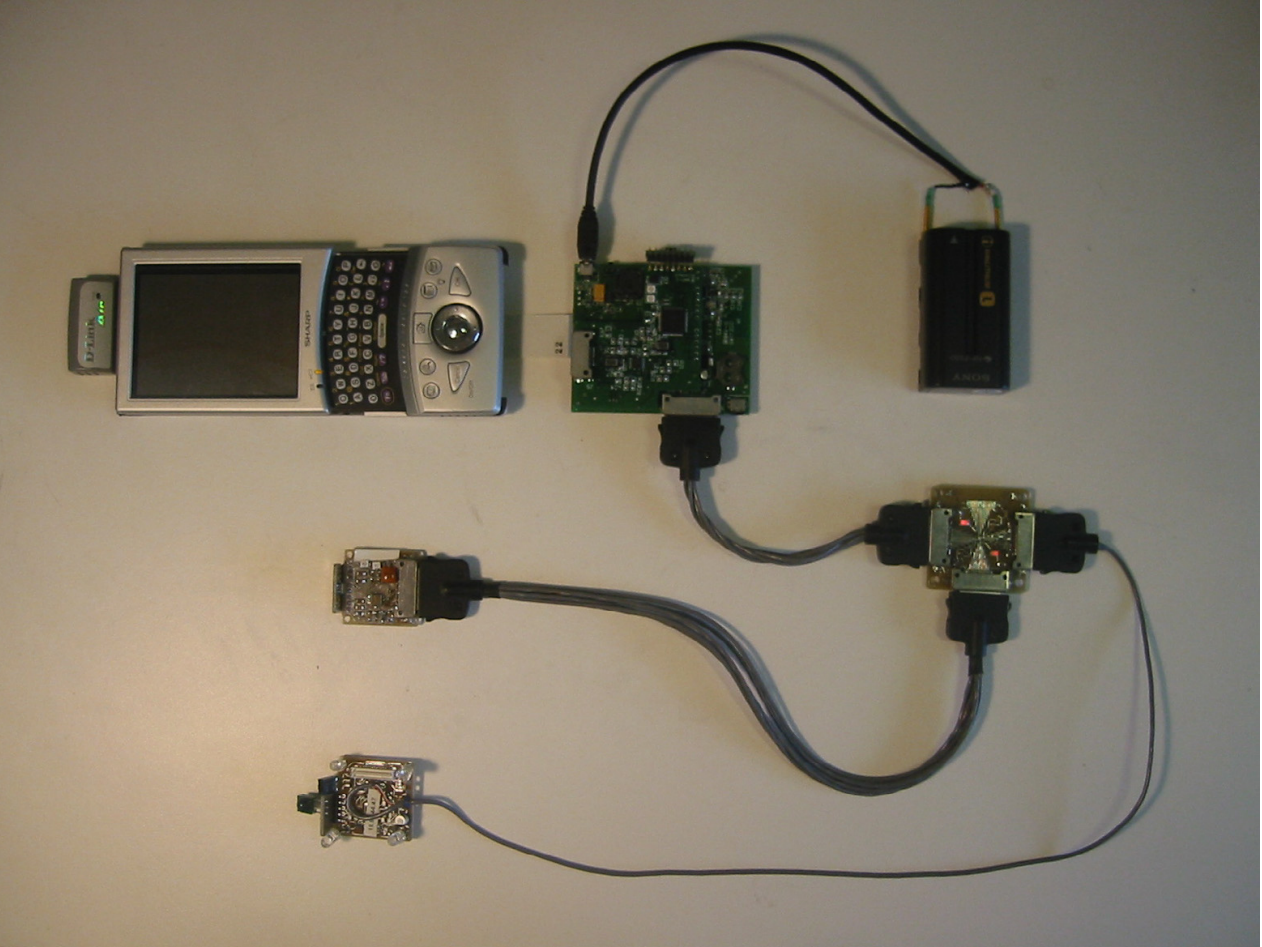
LiveNet system, composed of the Zaurus PDA (top left), with SAK2 data acquisition/sensor hub and BioSense physiological sensing board (middle), battery source (top right), sensor bus hub (lower right), 3D accelerometer board (middle left), and WMSAD multisensor board (lower left).

**Figure 3:**
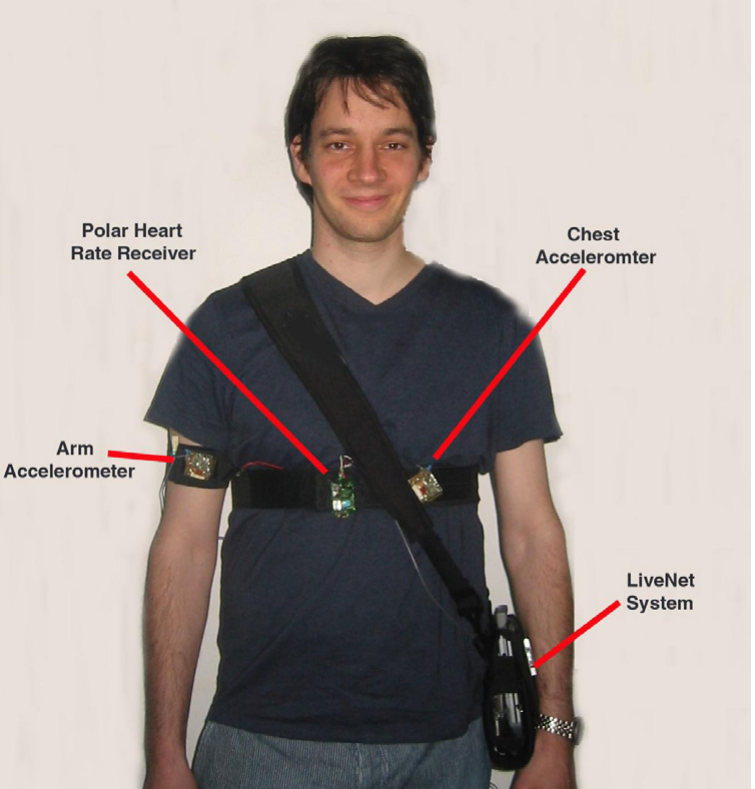
LiveNet wearable configured for non-invasive real-time soldier physiology monitoring.

**Figure 4:**
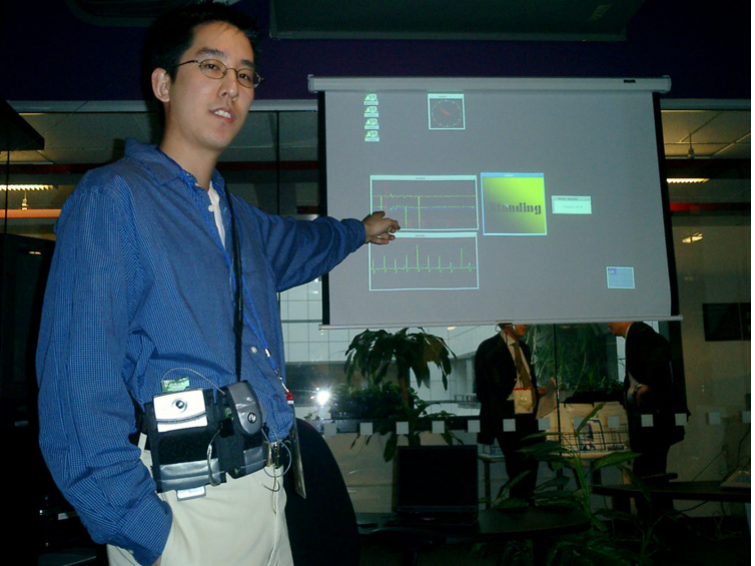
LiveNet wearable streaming real-time ECG/motion/stress information to a remote display over a wireless network link.
